# Cortical Ependymoma Presenting as Subclinical Seizures in a Very Young Child

**DOI:** 10.7759/cureus.37555

**Published:** 2023-04-14

**Authors:** Maya Prabhakaran, Babitha Sudarsanan, Gopalakrishnan C V, Zainul Aabideen

**Affiliations:** 1 Department of Pediatrics, Medeor 24x7 Hospital, Abu Dhabi, ARE; 2 Department of Pediatrics, LLH Hospital, Mussaffah, Abu Dhabi, ARE; 3 Department of Neurosurgery, Medcare Orthopaedics and Spine Hospital, Dubai, ARE; 4 Department of Pediatrics, Pediatric Hematology, and Oncology, Burjeel Medical City, Abu Dhabi, ARE

**Keywords:** chemotherapy, resection, subtle seizure, pediatric brain tumor, ependymoma

## Abstract

Supratentorial cortical ependymoma is an extremely rare malignancy in the pediatric population, especially in very young age groups. Most of the reported cases present with dramatic neurological symptoms like seizures and sudden onset hemiplegia. We hereby report a case of anaplastic supra-cortical ependymoma in a 13-month-old male child, with subtle seizures for four weeks. The child, who was brought for non-neurological complaints to the outpatient clinic, was found to have abnormal staring episodes. An electroencephalogram showed focal epilepsy and an MRI brain showed a large intra-axial lesion in the left frontal area. The child underwent gross total resection of the lesion and histopathology revealed WHO grade 3 cortical ependymoma.

## Introduction

Ependymomas are rare neuroectodermal tumors originating from the ventricular system. These are the third most common pediatric intracranial tumors, even though they account for only 1.2-7.8% of the total. About 90% of ependymomas are intracranial and among them, around one-third occur in the supratentorial compartment. Survival rates of posterior fossa tumors are inferior to supratentorial tumors. Surgical resection followed by radiotherapy has been the standard treatment recommendation, but recently chemotherapy has been suggested in very young children to avoid the detrimental effects of radiation on the developing brain [1].

## Case presentation

A 13-month-old boy, third born from a non-consanguineous marriage, was brought to the pediatric clinic for a general checkup and minor upper respiratory complaints. During history taking, the mother mentioned some abnormal behavior in the child which was noted four weeks earlier. The child had brief staring episodes lasting for around one minute, with a lack of response to sound or touch. According to the mother, multiple episodes were noted over the last four weeks. There was no loss of consciousness or tonic-clonic convulsions. The child did not have a fever and was active and interacting well. Since the parents did not consider it significant, they had not sought any medical attention. There was no family history of seizures, developmental disorders, or unexplained death.

On examination the child had normal vital signs, no neurocutaneous markers, and systemic examination was normal. There were no focal neurological signs, no facial deviation, and gait was normal.

**Figure 1:**
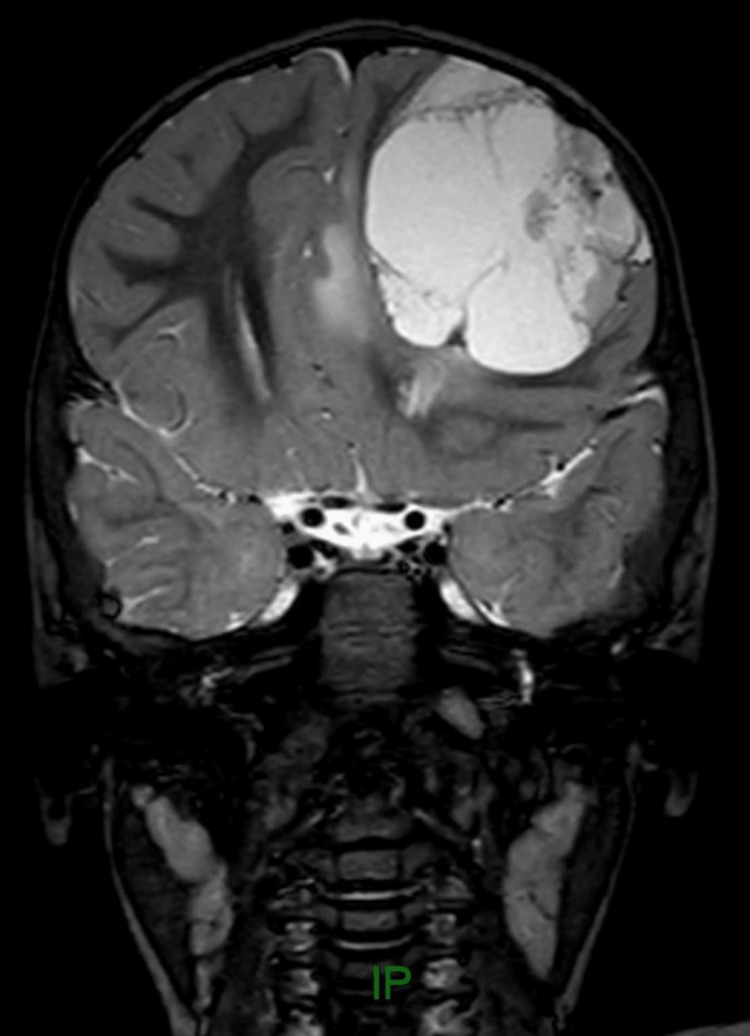
Pre-contrast coronal T2 image

**Figure 2:**
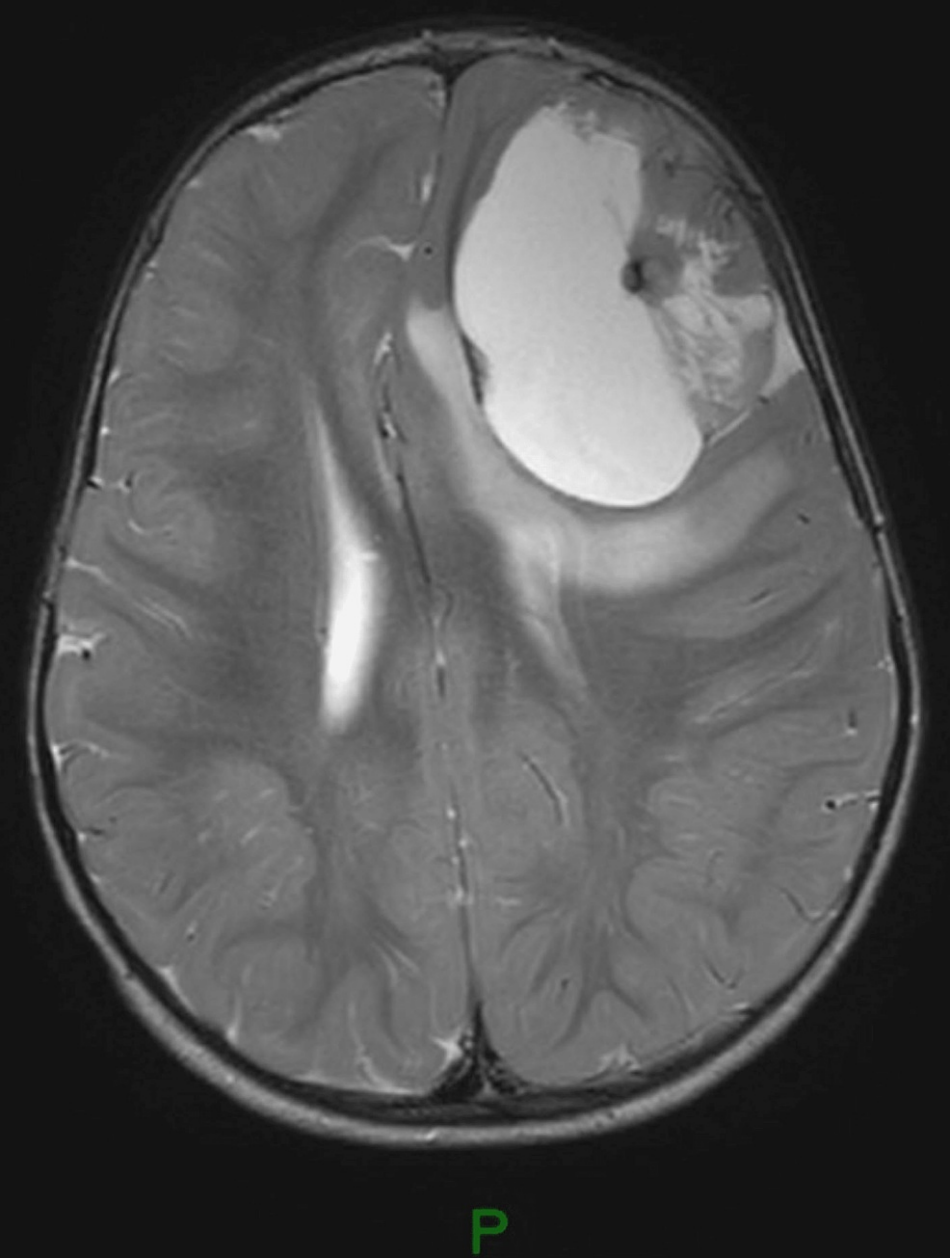
Pre-contrast axial T2 image

**Figure 3:**
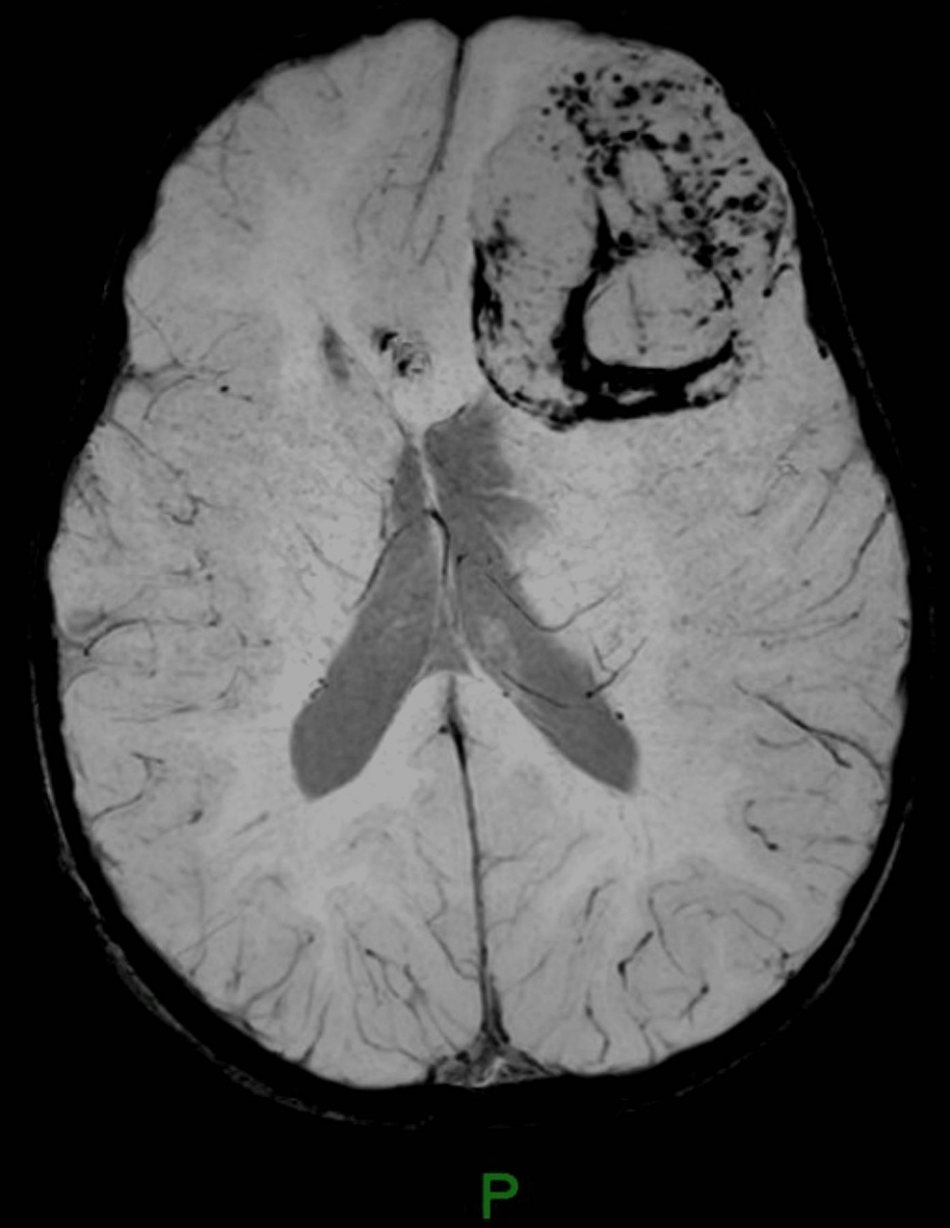
Susceptibility image demonstrating vascularity

**Figure 4:**
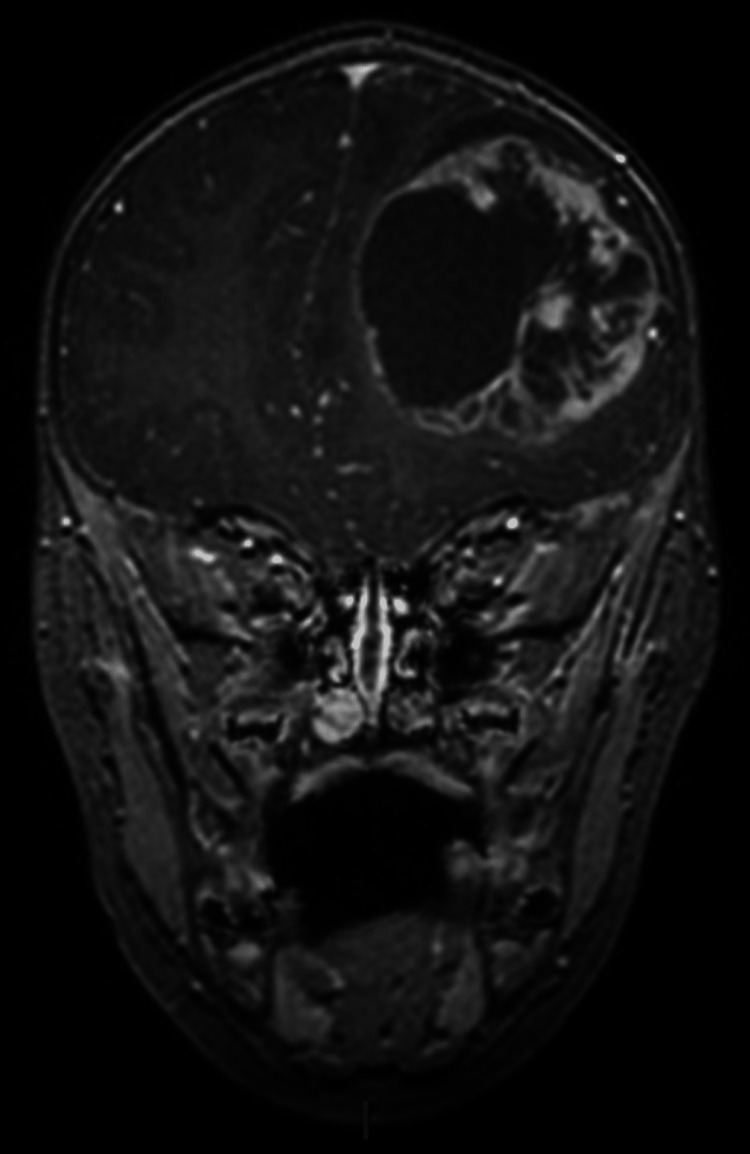
Post-contrast coronal T1 image

Suspecting epilepsy, the child was referred to a pediatric neurologist. The electroencephalogram (EEG) showed four episodes of electrographic seizures starting from the left frontal lobe and spreading to the left hemisphere, which suggested focal epilepsy. The child was started on oral valproic acid and further assessed by MRI brain. MRI brain showed a large intra-axial lesion in the left frontal lobe measuring 5.8x4.6x4.4 cm with peripheral contrast enhancement. MR spectroscopy showed high choline and low N-acetyl aspartic acid (NAA) peaks. There was perilesional edema with compression of the left frontal horn of the lateral ventricle and a midline shift of 8 mm to right. A differential diagnosis of supratentorial ependymoma or pilocytic astrocytoma was suggested (Figures 1-4).

**Figure 5:**
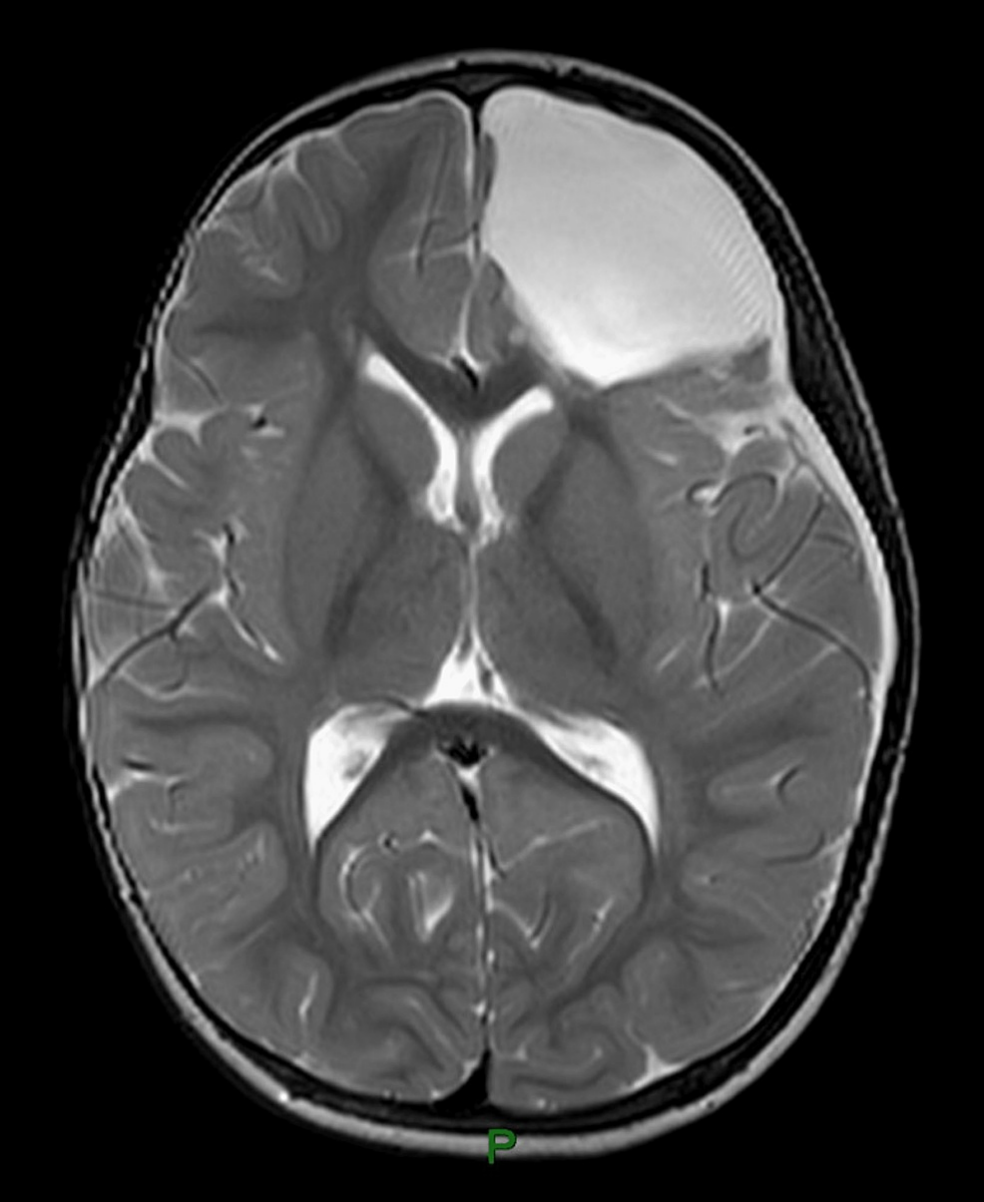
Postoperative axial T2 pre-contrast

**Figure 6:**
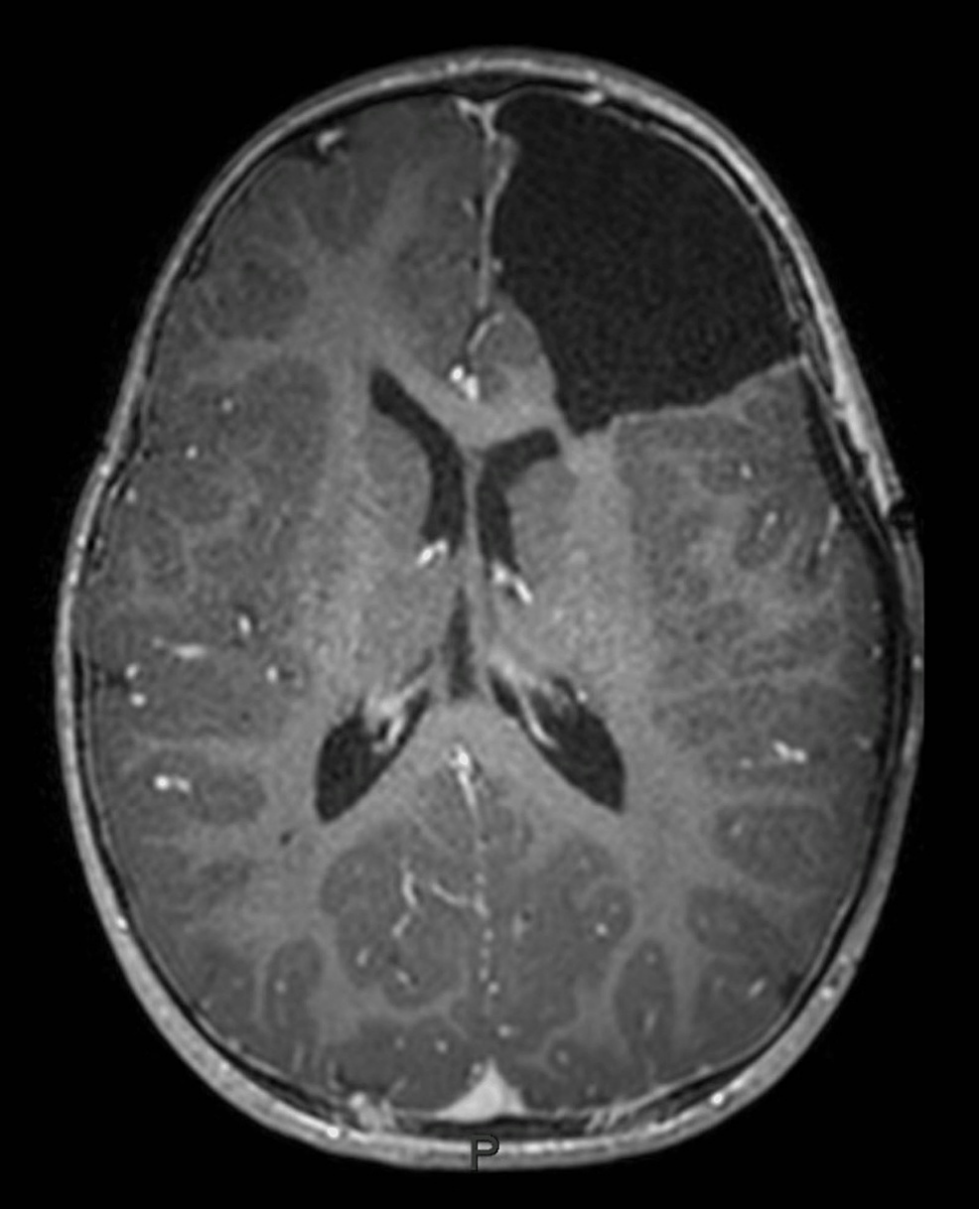
Postoperative axial T1 post-contrast

**Figure 7:**
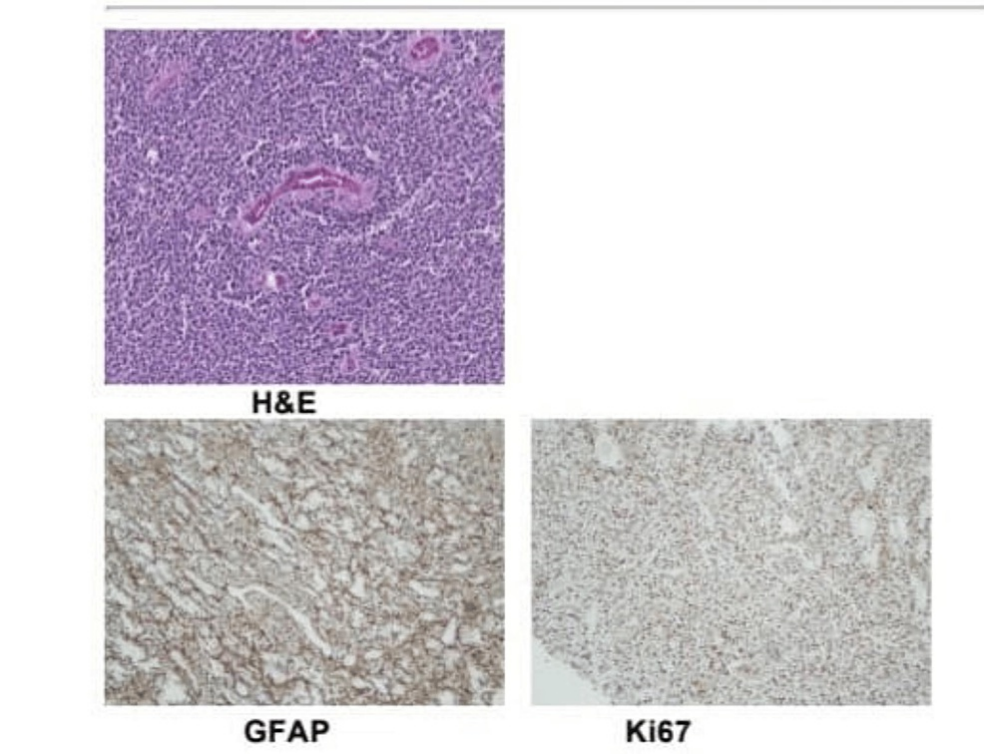
. Histopathology specimens demonstrating characteristic microscopic features, GFAP positivity, and high Ki 67 index H&E: hematoxylin and eosin staining

The child underwent surgery in the form of gross total excision of the tumor without any residual neurological sequelae (Figures 5, 6). The histopathology findings were consistent with anaplastic ependymoma, WHO Grade 3. The lesion had predominant round cell morphology with mild pleomorphism, hyperchromatic nuclei, and high nucleocytoplasmic ratio. Perivascular rosette formation was present and areas of hemorrhage were also noted. The tumor cells were positive for glial fibrillary acidic protein (GFAP), synaptophysin S 100, p53, and Olig2. Ki 67 index was 70-75% (Figure 7). Vimentin was not tested and epithelial membrane antigen (EMA) was negative. Positive GFAP, S 100, and synaptophysin were in favor of ependymoma. Olig 2 positivity is seen more in glial tumors, but may also be noted in glial tissue infiltration or injury. ATRX expression is retained in tumor cells and fluorescence in situ hybridization (FISH) studies were negative for deletion 11p/19q. Other molecular studies were not done in the specimen. 

The child is currently under the treatment of a pediatric oncologist for chemotherapy using cyclophosphamide, carboplatin, and etoposide. He is tolerating treatment well and has no neurological deficits. 

## Discussion

 Ependymomas are rare tumors of neuroectodermal origin. They arise from the ependymal lining of ventricles, choroid plexus, filum terminale, or central canal of the spinal canal. They account for 1.2-7.8% of all brain tumors [2]. In patients younger than 20 years, 60% of ependymomas are infratentorial, mainly arising from the fourth ventricle. Only 10-15% of cases occur in the first two decades of life and peak incidence is usually seen in the fourth and fifth decades of life [3]. Cortical ependymomas are a relatively uncommon subtype of ependymoma which is extra ventricular. These are uncommon in the pediatric population and have variable clinical courses depending on anatomical location and pathological grading [4]. They can appear at any age and do not have gender predilection. The symptoms may be very subtle relative to the size of the tumor and may be missed. Astrocytoma, ganglioma, oligodendroglioma, and other primitive neuroectodermal tumors are common differential diagnoses.

The clinical presentation depends on the age of the patient, tumor size, and location. The diagnosis is often made by neuroimaging, especially MRI. The MRI may show iso or hypointense lesions on T1 weighted images and hyperintense lesions on T2 weighted images. The lesion could be solid tumors with calcification necrosis or cystic changes. The lesions are usually well-circumscribed mass lesions. Calcification may be better appreciated in CT. MR spectroscopy may show elevated choline and low N-acetyl aspartate levels [5].

The ependymomas are classified according to the WHO grading system (updated in 2021). The WHO grading of ependymomas is based on features like pleomorphism, mitotic count, cellularity, vascularity, necrosis, and molecular and genetic features. Grade 1 is slow-growing myxopapillary and subependymal ependymoma, grade 2 is conventional ependymoma which is the most common form in the pediatric age group, and grade 3 is anaplastic ependymoma with aggressive tumor growth. The ependymoma cells have a positive reaction to glial fibrillary acid protein and protein S100 [6].

The treatment of ependymomas in children is based on surgical resection and radiation. The extent of the complete resection is an important prognosis factor. In the pediatric population, the majority of cases are infratentorial and may be complicated by incomplete resection whereas supratentorial lesions are more amenable to complete resection. The role of chemotherapy in children is still under debate but may be beneficial in very young children at risk of extensive radiation damage [7].

A recently published study by Cuoco et al. based on a systematic review of published cases of supratentorial ependymomas showed the mean age of presentation as 21.2 years in a reported number of 153 cases. Out of the total, 47 cases were of pediatric age groups, and only nine cases involved very young children less than three years [7]. Two other cases of supratentorial cortical ependymoma in very young children were reported by Mehdi et al. and Khilji et al. in 2016 and 2014, respectively [2,4]. The reports published in accessible journals were included in a comparison of clinical and radiological features.

Comparison of cases

We have done a comparison of cases involving very young patients of less than three years (Table 1). There were nine reported cases, the ninth one being the present case. In the reported cases, the male-to-female ratio was 2:1. There was only one patient under one year and our patient is the second youngest case reported. The most common presentation was hemiparesis (five out of nine), two with seizures alone, and one each with headache and vomiting. Our case presented with absence seizures and no focal neurological deficit. Right-sided lesions were more predominant than left-sided (2:1). Frontal region was most commonly affected.

**Table 1:** Comparison of cases in the literature involving patients younger than three years GTR: gross total resection. RT: radiotherapy

References	Age/gender	Location	Presentation	Lesion	WHO Grade	Treatment
Lehman (2008) [8]	2/F	Right temporal	Progressive left hemiparesis	6.5cm, partially cystic	3	GTR, RT
Lee et al. (2011) [9]	2/M	Right frontoparietal	Left leg clonic seizures	Solid lesion with calcification 1.5cm/1.5cm	2	GTR
Liu et al. (2014) [10]	2/M	Left frontal	Right hemiparesis	Solid tumor	3	GTR
Wang et al. (2020) [11]	0.75/M	Right frontal	Vomiting	Solid/cystic tumor	3	GTR
	2/F	Right frontal	Headache	Cystic tumor	3	GTR, RT
	2/M	Left frontoparietal	Hemiparesis	Cystic tumor	3	STR
Darmoul et al. (2016) [2]	1.3/F	Right frontopareital	Lt hemiparesis, seizures	Solid cystic tumor7.6/7cm	3	GTR
Khilji et al. (2014 )[4]	2/M	Right frontotemporal	Headache, left hemiplegia, and seizures	Solid and cystic mass	3	GTR, RT
Present case	1/M	Left frontal	Subtle seizures	Solid cystic lesion 5.8/4.6cm	3	GTR, CT

## Conclusions

Supratentorial cortical ependymomas are rare in very young children. Large supratentorial tumors can present with minimal signs and symptoms. Gross complete resection with or without radiation is the cornerstone of treatment. Cortical ependymomas have a slightly more favorable prognosis than other supratentorial ependymomas. A high index of suspicion is recommended in evaluating very young children with abnormal behavior or movements noted by parents. The present case is an example of a large lesion with a midline shift presenting with a history suggestive of subtle seizures in an otherwise normal infant.
